# Reordering for Improved Constrained Reconstruction from Undersampled k-Space Data

**DOI:** 10.1155/2008/341684

**Published:** 2008-12-11

**Authors:** Ganesh Adluru, Edward V. R. DiBella

**Affiliations:** ^1^Laboratory for Structural NMR Imaging, Department of Radiology, University of Pennsylvania, Philadelphia, PA 19104, USA; ^2^Electrical and Computer Engineering Department, University of Utah, Salt Lake City, UT 84112, USA; ^3^Utah Center for Advanced Imaging Research, Department of Radiology, University of Utah, Salt Lake City, UT 84108, USA; ^4^Department of Bioengineering, University of Utah, Salt Lake City, UT 84112, USA

## Abstract

Recently, there has been a significant interest in applying reconstruction techniques, like constrained reconstruction or compressed sampling methods, to undersampled k-space data in MRI. Here, we propose a novel reordering technique to improve these types of reconstruction methods. In this technique, the intensities of the signal estimate are reordered according to a preprocessing step when applying the constraints on the estimated solution within the iterative reconstruction. The ordering of the intensities is such that it makes the original artifact-free signal monotonic and thus minimizes the finite differences norm if the correct image is estimated; this ordering can be estimated based on the undersampled measured data. Theory and example applications of the method for accelerating myocardial perfusion imaging with respiratory motion and brain diffusion tensor imaging are presented.

## 1. INTRODUCTION

There has been large interest in speeding the acquisition of MRI data by acquiring fewer samples in *k*-space and resolving the artifacts. Recently, there have been significant advances in applying inverse problem techniques to reconstruct images from undersampled *k*-space MRI data [1–6]. The methods use nonuniform undersampling and a nonlinear recovery scheme in which a constraint, such as a spatial total variation (TV) constraint [7], is applied on the estimated solution, while preserving fidelity to the acquired data in *k*-space. It has been shown [2] that using an *L*_1_ norm or a TV norm as constraint exploits the implicit sparsity in the data, and can be used in both space and time dimensions. The method is best known to reconstruct piecewise constant or smoothly varying data from its undersampled Fourier samples; but the application of the method to MR imaging techniques like dynamic contrast enhanced myocardial perfusion imaging (with respiratory motion in the data) and diffusion tensor imaging (DTI) can be limited as these images are often not piecewise constant.

In this paper, we propose a technique to improve the reconstruction of general signals that may not fit the TV constraint well. The technique uses preprocessing of the measured undersampled data to determine an improved ordering of the pixel intensities of the image estimate. If an ordering that improves the match of the estimated images and the constraint being used within the reconstruction can be found, an improved reconstruction can result. The image estimates are reordered solely to be used with the constraint or regularization term in the iterative reconstruction. The reordering approach is general in its applicability and can be used in contexts which are based on regularization techniques and in which ordering of the image intensities can be determined a priori. In the next sections, we give a brief overview of the compressed sampling or constrained reconstruction method for MRI from a regularization point of view and then present the theory and applications of the reordering method.

## 2. THEORY

### 2.1. Compressed sampling/constrained reconstruction method

The compressed sampling method is described rigorously from a mathematical standpoint recently in a series of papers [2, 8–10]. The method is used to reconstruct a signal from a set of random Fourier samples below the Nyquist rate by solving a convex optimization problem, in which fidelity to the measured data is preserved at sample locations, while applying an *L*_1_ or a TV constraint on the estimated solution. The method exploits the implicit sparsity in the estimated solution or a transform of the estimated solution. One useful transform for signals or images is finite differences as the signals that are not directly sparse can be sparse in terms of finite differences (especially piecewise constant or smoothly varying signals or images) and hence an *L*_1_ norm of finite differences (TV norm) is used.

Although the *L*_1_ norm of the signal or image estimate is not a direct measure of sparsity in the data, it has been shown that for a wide variety of data using an *L*_1_ norm is equivalent to using the *L*_0_ norm (the number of nonzero samples), which is a direct measure of sparsity [2]. Solving the optimization problem with an *L*_1_ norm is generally easier than solving the problem with an *L*_0_ norm.

The compressed sampling method when applied to MR image reconstruction can be thought as a constrained reconstruction method in an inverse problem framework [3, 11–14]. For the 1D case, the relation between Fourier data and the signal space estimate can be represented as *F* *m* = *d*, where *m* is the signal of interest, *d* is the fully sampled *k*-space data, and *F* represents the Fourier transform; but it often takes a long time to acquire full *k*-space data and results in tradeoffs in image quality, resolution, and coverage of the organ. To accelerate the data acquisitions, when full data are not acquired in *k*-space, and only undersampled data are acquired, the relation between the artifact-free signal estimate $\tilde{m}$ and acquired data is given by

(1) $$WF\tilde{m}\;=\;\tilde{d},$$

where *W* implements a binary undersampling pattern with ones (where data are acquired) and zeros (where data are missing), and $\tilde{d}$ is the undersampled Fourier data. Reconstructing the signal $\tilde{m}$ directly using (1) is not feasible as *W* ^−1^ does not exist in general and hence the solution is not unique.

Regularization techniques can be used to solve this ill-posed problem. The existence of the solution is imposed by considering least-square solutions which minimize the functional ${∥WF\tilde{m}\;-\;\tilde{d}∥}_{2}^{2}$, where ||·||_2_ represents an *L*_2_ norm. Uniqueness of the solution is imposed by using one or more constraints on the solution. A popular constraint used in the field of compressed sampling is the total variation constraint given by ${∥\sqrt{\nabla {\tilde{m}}^{2}\;+\;\varepsilon }∥}_{1}$, where ∇ is the gradient of the estimated signal, *ε* is a small positive constant to avoid singularities in the derivative of the functional [15], and ||·||_1_ represents an *L*_1_ norm.

Reconstruction is performed by minimizing a convex cost function (*C*)

(2) $$C\;=\;{∥WF\overset{˜}{m}\;-\;\overset{˜}{d}∥}_{2}^{2}\;+\;\alpha {∥\sqrt{\nabla {\overset{˜}{m}}^{2}\;+\;\varepsilon }∥}_{1}.$$

Hence, a solution which preserves fidelity to the acquired data and which has the minimum total variation is chosen as the final solution. In (2), *α* is the regularization parameter which controls the tradeoff between the fidelity and the constraint terms. The total variation constraint helps to resolve the artifacts while not penalizing the edges heavily.

The method can be extended to 2D and multi-image dimensions and it works very well when the *k*-space data are undersampled in an irregular fashion and the underlying complex images are smoothly varying or are piecewise constant [2]. When the images are not piecewise constant (which is the case for most MR images), the performance of the method can be affected. We describe below a reordering method to improve the performance of the constrained reconstruction method when the data do not match the constraints well. The method preprocesses the signal to select a monotonic ordering of the estimated solution in space and/or time and incorporates the reordering in the constraints to obtain better reconstructions.

For clarity, the reordering method is first described for the 1D case and then the method is extended to 2D and multidimension cases. Applications of the reconstruction method with reordering for dynamic myocardial perfusion imaging with respiratory motion and for brain DTI data are presented.

### 2.2. Reordering method: 1D case

**Figure 1:**
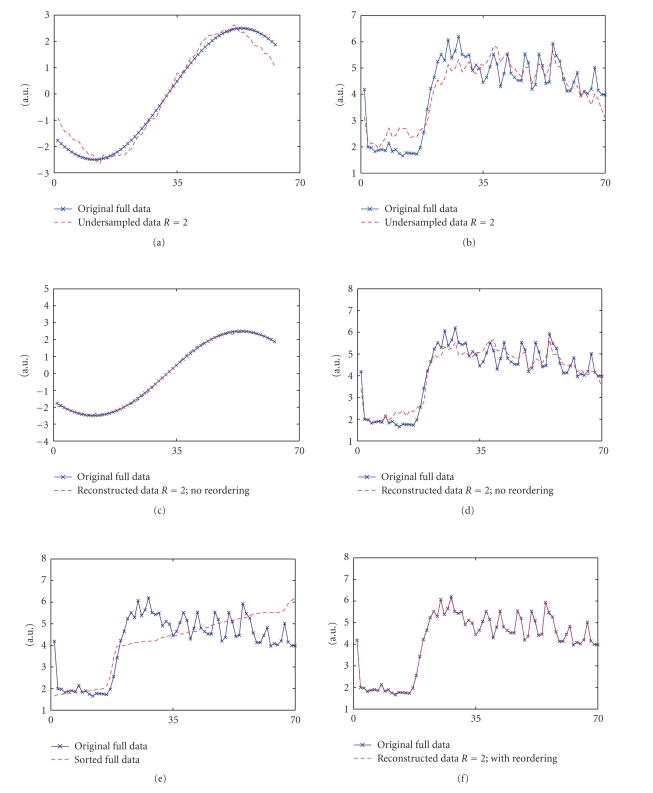
(a) A fully sampled smoothly varying 1D signal and the corresponding signal reconstructed using IFT from its incomplete Fourier data undersampled by a factor of two (*R*~2) in a random fashion. (b) A fully sampled nonsmooth varying 1D signal and the corresponding signal reconstructed using IFT from its *R*~2 Fourier data undersampled in a random fashion. (c) Comparison of the original fully sampled smooth signal and the reconstructed signal from *R*~2 Fourier data without reordering. (d) Comparison of the original fully sampled nonsmooth signal and the corresponding signal reconstructed from *R*~2 Fourier data without reordering. (e) Comparison of the original fully sampled nonsmooth signal and the corresponding sorted signal. (f) Comparison of the original fully sampled nonsmooth signal and the signal reconstructed from *R*~2 Fourier data with reordering.

When the signal of interest is varying rapidly and is not smooth or the data are not piecewise constant, the total variation of the signal is already high and hence reconstruction from undersampled Fourier domain samples can be inaccurate. Consider, for example, a smoothly varying 1D signal and a rapidly varying signal that are labeled “original full data” as shown in Figures 1(a) and 1(b), respectively. When the corresponding Fourier samples (*k*-space data) of the curves are undersampled by a factor of two in a pseudorandom fashion (using “rand” function in MATLAB (The Mathworks, Natick, Mass, USA)) and reconstructed using the inverse Fourier transform, the signals labeled “undersampled data *R* = 2” in Figures 1(a) and 1(b) are obtained. When these undersampled signals are reconstructed according to (2), the curves in Figures 1(c) and 1(d) are obtained. The original curves are overlaid in Figures 1(c) and 1(d) for reference.

Reconstruction using (2) is better for the smooth curve in Figure 1 as compared to that for the rapidly varying curve. To improve the reconstruction in the latter case, we first reorder the estimated curve in the signal space according to an optimal order, and then apply the total variation constraint. The optimal ordering can be determined as the ordering that makes the signal intensities in the curve from the fully sampled dataset monotonic and smoothly varying. Reordering the estimated solution helps by reducing sudden variations in the curves and gives a better match to the assumed constraint. In practice, the curve or images from fully sampled data will not be available to obtain the optimal ordering and some sort of approximate reconstruction must be used to determine the ordering. While this area needs more research, we show here that relatively simple methods for determining reorderings can improve reconstructions of some types of undersampled data.

Better reconstruction from the undersampled Fourier samples is obtained when the reordered curve is used in the constraint term, as the a priori assumption that the curve has lower variation is better satisfied. So the new reconstruction from undersampled data is performed according to (3) in which the only difference is that the TV constraint is applied on the reordered data as opposed to applying the constraint directly on the given data. Reordering the estimated signal can also be thought as multiplication of the signal with a reordering matrix “*P*.” This matrix can be a permutation matrix of ones and zeros (it could also be a diagonal matrix for a 1D signal and it can be generalized for multidimensional signals) as follows:

(3) m^ = minm˜∥WFm˜ − d˜∥22 + α∥∇(Pm˜)2 + ε∥1.

Note that the reordering in (3) is not directly based on the intensity values obtained from the aliased signal from undersampled data in *k*-space (or for other applications, whatever domain the measurement data is obtained). That is, *P* is determined once, and is fixed while minimizing (3). Ordering the undersampled data according to the optimal order does not mean that the reordered undersampled image estimates are monotonic, but means that if this ordering is used, the original full data in the signal space will best match the TV constraint. Consider Figure 1(e) which shows the sorted curve of the original curve in Figure 1(b). The curve is monotonic and smoothly varying and has lower total variation as compared to the original curve.

The reconstruction obtained with reordering is shown in Figure 1(f). The ordering in this case was chosen as the sorting order that made the original full curve monotonic and smoothly varying. The reconstructed and the original signals match very closely. Although not shown here, the reconstruction with reordering was comparable to that without reordering for the case of the smooth curve in Figure 1(a).

**Figure 2:**
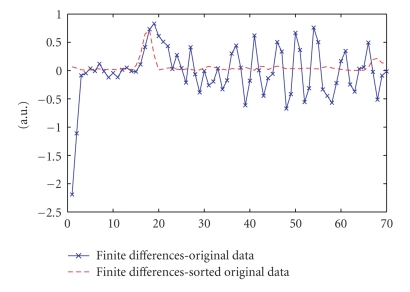
Comparison of sparsity of the fully sampled original nonsmooth signal in Figure 1(b) and that of the corresponding sorted signal in terms of finite differences.

From the compressed sampling point of view, reordering the data can lead to sparser representations of the data and hence higher acceleration factors. Alternatively, better reconstructions for a given acceleration factor can be obtained. Figure 2 illustrates the point for the original full curve shown in Figure 1(b). The figure compares the sparsity of the original curve and the sorted curve in terms of finite differences. The finite difference curve for the sorted signal is sparser (has fewer nonzero values) as compared to that of the original signal and hence using an *L*_1_ norm of the finite differences with reordering leads to better reconstructions.

### 2.3. Choice of the regularization parameter, *α*

**Figure 3:**
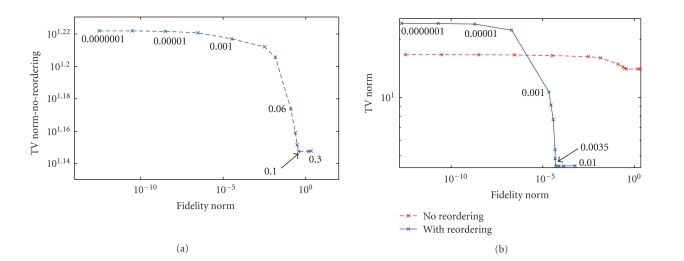
Comparison of optimal regularization weights without and with reordering. *L*-curves obtained for reconstruction of the nonsmooth signal in Figure 1(b) from *R*~2 Fourier data (a) without reordering and (b) with reordering overlaid by the *L*-curve in Figure 3(a).

Choosing the optimal regularization parameter *α* is important to obtain good reconstructions. The *L*-curve method [16] is a popular technique for choosing the optimal value. The method can be used when reordering is used. The fidelity norm is plotted against the constraint norm of the reordered data and the optimal parameter is given by the corner of the *L*-curve. Figures 3(a) and 3(b) show the *L*-curves obtained for reconstructions from undersampled data for the curve shown in Figure 1(b) without and with reordering, respectively. The *L*-curve in Figure 3(a) is also overlaid on Figure 3(b) for direct comparison. The *L*-curves and the optimal parameters obtained are different for both cases. The optimal parameter without reordering is higher than that with reordering. As in the iterative methods, the number of iterations also plays the role of regularization parameter; a fixed maximum number of iterations which gave minimum RMS reconstruction error for various *α* values was chosen in computing the *L*-curves.

### 2.4. Inaccurate reordering

From the above, it is apparent that correct reordering can help in better reconstruction from undersampled Fourier data when the signals are not smoothly varying. In the above experiment, we used full data to determine the optimal ordering. To be able to use the reordering method, we need to have an ordering that makes the original signal best match the constraint. In practice, it is likely not possible to get the exact ordering of the signal curves or images as that obtained using fully sampled Fourier data due to various factors like blurring of the prior signal, noise in the prior signal, and so on. To simulate this case, we randomly perturbed the exact sorting order to see the effect of having inexact ordering on the performance of the algorithm.

**Figure 4:**
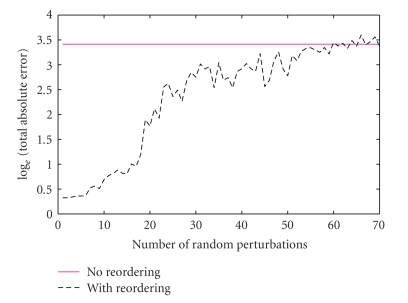
Comparison of errors in the reconstruction for the nonsmooth signal in Figure 1(b) without reordering and with reordering as a function of inaccuracies in the ordering.

In Figure 4, the *X*-axis represents the number of random perturbations, that is, the number of indices of the exact “sorting-order vector” that are randomly perturbed. Consider **S** to be the sorting-order vector for the original signal. When there is one random perturbation, (i) a random number is generated between 1 and the length of **S** denoted by r_a, then (ii) a second distinct random number between 1 and the length of **S** denoted by r_b, and finally (iii) the value of the **S** at index r_a is exchanged with that at index r_b. A value of 10 on the *X*-axis means that the values of the exact “sorting-order vector” at 10 distinct randomly picked indices (out of 70) are exchanged with those at a different set of 10 distinct randomly picked indices. The *Y*-axis represents the natural log of the total absolute difference between original full data and the reconstructed signal. We can see that as the number of random perturbations increases, the total absolute error using reordering gradually increases, but this number is still better or comparable to that without the reordering except for a few perturbations toward the end of the plot where the entire sorting-order vector was randomly perturbed.

### 2.5. Reconstruction with reordering: 2D case

**Figure 5:**
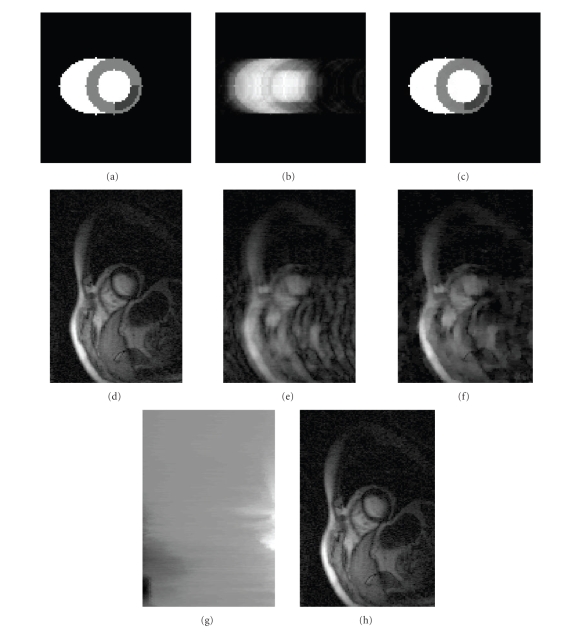
Reordering method for 2D images. (a) Simulated piecewise constant heart image. (b) Image reconstructed using IFT from ~15% of the full Fourier data, undersampled in a variable density random fashion. (c) Image reconstructed from undersampled data using a TV spatial constraint. (d) Actual MR magnitude image of the short-axis slice of a heart at a single point in a perfusion sequence reconstructed from fully sampled *k*-space data using IFT. (e) Corresponding IFT reconstruction from *R*~3 *k*-space data undersampled in VD random fashion. (f) Reconstruction using a 2D TV constraint without any reordering. (g) Row-reordered image of the real part of the complex MR image of the heart. (h) Reconstructed image with spatial reordering using a TV constraint. Ordering of the data here was obtained using the image reconstructed from fully sampled data.

The reordering method described above for the 1D case can be extended to 2D and applied in the context of images. As in the 1D case, reordering in 2D for images helps in better reconstruction when the images of interest are nonsmooth or are not piecewise constant. For example, Figure 5(a) shows a simulated piecewise constant heart image with blood pools and with an ischemic region in the myocardium. When full Fourier data of the image are undersampled in a variable density (VD) Cartesian fashion [5] (so that 5 lines in the center of *k*-space are fully sampled and the remaining phase encodes are sampled in a pseudorandom fashion to give a net reduction factor of ~6.5), direct inverse Fourier transform reconstruction gives the image shown in Figure 5(b). When the constrained reconstruction approach with a TV spatial constraint (${∥\sqrt{\nabla_{x}{\tilde{m}}^{2}\;+\;\nabla_{y}{\tilde{m}}^{2}\;+\;\varepsilon }∥}_{1}$) without reordering is used, Figure 5(c) is obtained. We can see that Figure 5(c) matches well with Figure 5(a); but when the image is not piecewise constant, the performance of the constrained reconstruction can be affected. Figure 5(d) shows an actual MR heart image from a patient at a single time in a perfusion sequence. The image was reconstructed using a standard 2D inverse Fourier transform of the fully acquired *k*-space data. Figure 5(e) shows the standard 2D inverse Fourier transform reconstruction of undersampled *k*-space data for the time frame, with zeros inserted for the missing *k*-space data points. The data were undersampled by a factor of three in VD Cartesian fashion (10 phase encodes fully sampled around the center and the remaining ones in a pseudorandom fashion). Figure 5(f) shows the constrained reconstruction from the undersampled data using a spatial TV constraint without any reordering which has a few residual artifacts. For improving the reconstruction in this case, the image is reordered independently in *x* and *y* directions before applying the 2D TV constraint, that is, reorderings are determined separately for each row and each column. In practice, since the data we deal with in MRI are complex, the optimal ordering is determined independently for the real and imaginary components of the image and separately in *x* and *y* directions. A row-reordered real part of the image of the original complex MR image of the heart is shown in Figure 5(g). The TV spatial constraint with reorderings for a complex image can be explicitly written as ${∥\sqrt{𝒴}∥}_{1}$, where $𝒴\;=\;\nabla_{x}{\left(P_{Rx}{\overset{˜}{m}}_{\text{Real}}\right)}^{2}\;+\;\nabla_{x}{\left(P_{Ix}{\overset{˜}{m}}_{\text{Imag}}\right)}^{2}\;+\;\nabla_{y}{\left(P_{Ry}{\overset{˜}{m}}_{\text{Real}}\right)}^{2}\;+\;\nabla_{y}{\left(P_{Iy}{\overset{˜}{m}}_{\text{Imag}}\right)}^{2}\;+\;\varepsilon$, in which ${\tilde{m}}_{\text{Real}}$ is the real part of the image estimate and ${\tilde{m}}_{\text{Imag}}$ is the imaginary part of the image estimate. *P_Rx_* and *P_Ix_* denote the reordering matrices for ordering the signal in *x* dimension for the real and imaginary parts, respectively, while *P_Ry_* and *P_Iy_* are the corresponding reordering matrices in *y* dimension. For simplicity and compactness, the above spatial constraint with reordering is referred to as ${∥\sqrt{\nabla_{x}{\left(P_{x}\overset{˜}{m}\right)}^{2}\;+\;\nabla_{y}{\left(P_{y}\overset{˜}{m}\right)}^{2}\;+\;\varepsilon }∥}_{1},$ where $P_{x}\tilde{m}$ gives the image reordered for each row in the *x* direction and $P_{y}\tilde{m}$ is the image reordered for each column in the *y* direction. Figure 5(h) shows the reconstruction from the undersampled data using a spatial TV constraint with reordering. The ordering of the data was obtained using the image reconstructed from fully sampled data.

**Figure 6:**
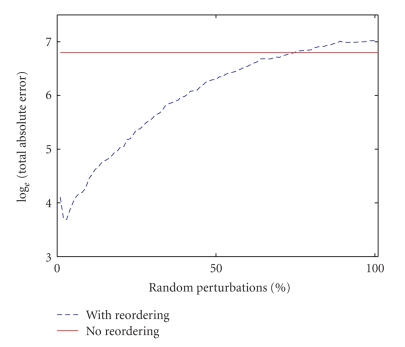
Comparison of errors in the reconstruction for the actual MR heart image in Figure 5(d) without reordering and with reordering as a function of perturbations in the exact spatial ordering.

Figure 6 shows a plot comparing the reconstruction error with increasing number of perturbations in the exact spatial ordering for the actual MR heart image in Figure 5(d). The reconstruction error is calculated as the total absolute difference between the full data reconstruction and the data reconstructed using the TV spatial constraint. A value of 10 on the *X*-axis means that the sorting-order vectors for 10% of the total number of rows (rounded to nearest integer and randomly picked) and those for 10% of the total number of columns (rounded to nearest integer and randomly picked) are randomly perturbed for both the real and imaginary parts of the complex image data.

Perturbation for a given row or a column is done independently for the entire length of the sorting-order vector as described for the 1D case in Section 2.4. A value of 100 on the *X*-axis means that the sorting-order vectors for all the rows and all the columns are completely perturbed (randomly and independently) for real and imaginary parts of the image. The error for the reconstruction with reordering is gradually increasing with increasing perturbations and when the exact sorting orders are severely modified, the error gets higher than that without reordering.

### 2.6. Reordering: multiple dimensions

The reordering method described above can be extended to multi-image MR acquisitions like dynamic myocardial perfusion imaging and brain DTI. In perfusion imaging, a series of images of the heart are acquired to track the uptake and washout patterns of the contrast agent in the myocardium. DTI requires the acquisition of multiple images with diffusion weightings in different directions. Reordering can be done in the multi-image dimension—in the time dimension for the case of myocardial perfusion imaging and in the diffusion encoding dimension for the case of DTI. As in the 1D case, reordering in the multi-image dimension for the images can give a better reconstruction when the signal changes in the dimension are not smoothly varying which is the case for perfusion imaging with respiratory motion and for DTI. The constraint for the reordering in the multi-image dimension is represented as ${∥\sqrt{\nabla_{t}{\left(P_{t}\overset{˜}{m}\right)}^{2}\;+\;\varepsilon }∥}_{1},$ where ∇_*t*_ represents the gradient operator in the multi-image dimension and $P_{t}\tilde{m}$ is the data reordered in the corresponding dimension. The subscript *t* is used because the multi-image dimension is analogous to the temporal dimension of dynamic perfusion datasets. For a given image frame in the multi-image dataset, 2D spatial reordering can also be included as described in Section 2.4.

Reconstruction can then be performed by using TV constraints in both space and multi-image dimensions and with reordering in the corresponding dimensions as follows:

(4) $$C\;=\;{∥WF\overset{˜}{m}\;-\;\overset{˜}{d}∥}_{2}^{2}\;+\;\alpha_{1}{∥\sqrt{\nabla_{t}{\left(P_{t}\overset{˜}{m}\right)}^{2}\;+\;\varepsilon }∥}_{1}+\;\alpha_{2}{∥\sqrt{\nabla_{x}{\left(P_{x}\overset{˜}{m}\right)}^{2}\;+\;\nabla_{y}{\left(P_{y}\overset{˜}{m}\right)}^{2}\;+\;\varepsilon }∥}_{1}.$$

A similar framework to (4) was proposed in [12] for reconstructing undersampled radial myocardial perfusion data but without reordering and with a different temporal constraint. As in [12], (4) will be referred to as a spatio-temporal constrained reconstruction (STCR). 

## 3. APPLICATIONS

### 3.1. Dynamic phantom data

The reordering method for multi-image acquisitions (4) was tested using a dynamic phantom. Gd was slowly injected into a tube running through a water phantom and fully sampled Cartesian *k*-space data were acquired over time using an echoplanar imaging sequence on a Siemens 3T Trio scanner. Raw *k*-space data was then undersampled offline in a variable density (VD) pseudorandom fashion, in which 12 central low-resolution *k*-space lines were sampled for all time frames, and the remaining phase encodes were sampled in a pseudorandom fashion to give a net reduction factor of three. The acquisition matrix for the scan was 256 × 72. Reconstruction from the undersampled data was then performed according to (4) in two steps. In the first step, the information about the reordering was obtained using images obtained using the central low-resolution data from VD undersampling. The image estimates were reordered first in the time dimension only and the reconstruction was performed. In this step, the real and imaginary parts of the complex low-resolution image space data for each pixel were sorted independently in the time dimension according to their intensity values. The corresponding sorting orders for the real and imaginary parts were used for reordering the real and imaginary parts of the complex undersampled image space data. After performing an initial reconstruction with only temporal reordering, the resulting data were used to determine the spatial ordering. Final reconstruction was then performed using spatial and temporal reordering. Results of the final reconstruction with reordering were compared to full data reconstructions using the standard inverse Fourier transform and to the reconstructions without any reordering.

### 3.2. Dynamic myocardial perfusion and brain DTI data

The reordering method was applied on dynamic myocardial perfusion imaging with respiratory motion and on brain DTI data. Full Cartesian raw *k*-space perfusion data were obtained using a Siemens 3T Trio scanner using a TurboFLASH saturation recovery sequence. The parameters for the data acquisition were TR = 1.8 milliseconds, TE = 1 millisecond, flip angle = 12°, Gd dose = 0.025 mmol/kg, slice thickness = 6 mm, and acquisition matrix = 192 × 96. FOV = 380 × 285 mm^2^. The data were acquired with informed consent in accordance with the University of Utah Institutional Review Board. Brain DTI image data were acquired on a GE 3T Scanner and full *k*-space data were generated from the magnitude image data by applying 2D Fourier transforms on each diffusion encoding direction. Full *k*-space data for both perfusion and brain DTI data were undersampled in a variable density pseudorandom fashion outside the center and with the central 18 *k*-space lines sampled for each time frame, and reconstruction was performed in two steps as described in Section 3.1. The net *R* value for the perfusion data was 2.5 while that for the DTI data was 3.

## 4. RESULTS

### 4.1. Dynamic phantom data

**Figure 7:**
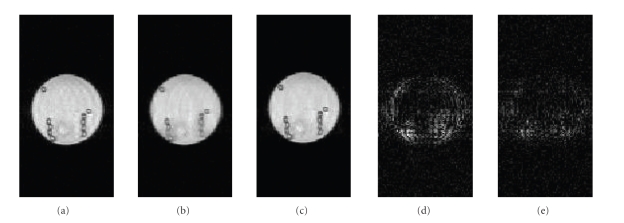
Results of multi-image reordering method on dynamic phantom data. Image at a time point reconstructed (a) from full *k*-space data using IFT, (b) from *R*~3 data using STCR without any reordering, and (c) from *R*~3 data using STCR with reordering in time and spatial dimensions. (d) Absolute difference image between Figures 7(a) and 7(b). (e) Absolute difference image between Figures 7(a) and 7(c).

The results of the reordering method on the multi-image phantom data are shown in Figure 7. Figure 7(a) shows the image of a slice that was reconstructed from full data using IFT at single time point. Figure 7(b) shows the corresponding image reconstructed from *R*~3 data using STCR without any reordering. Figure 7(c) shows the image reconstructed from *R*~3 data using STCR with reordering in both temporal and spatial dimensions.

Figure 7(d) shows the absolute difference image between Figures 7(a) and 7(b) and Figure 7(e) shows the absolute difference image between Figures 7(a) and 7(c). The images in Figures 7(d) and 7(e) are scaled to the same window level to highlight the differences. Figure 7(d) has more structure as compared to Figure 7(e).

### 4.2. Dynamic myocardial perfusion

**Figure 8:**
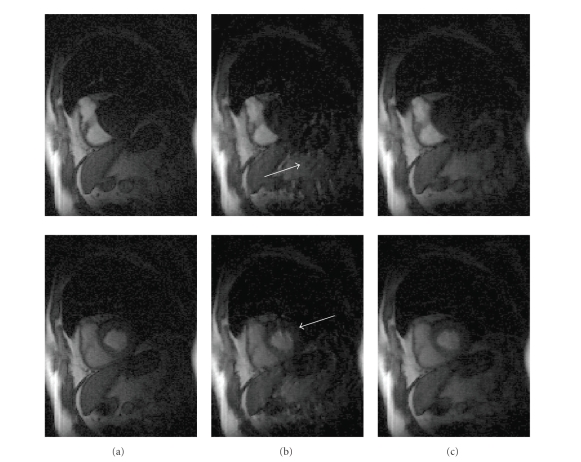
Result of multi-image reordering method on dynamic myocardial perfusion data. (a) Images at two different time points in the sequence reconstructed from full *k*-space data (first column). (b) Corresponding images reconstructed from *R*~2.5 *k*-space data, undersampled in variable density random fashion, using constrained reconstruction method in (4) but without any reordering (second column). The arrows point to the residual artifacts in the images. (c) Corresponding images reconstructed from *R*~2.5 *k*-space data using constrained reconstruction method in (4) with reordering (third column).

The results of the reordering method for dynamic myocardial perfusion imaging are shown in Figure 8. Figure 8(a) (column) shows images at two different time points in a perfusion sequence reconstructed from full perfusion data using standard inverse Fourier transforms. Figure 8(b) (column) shows the corresponding STCR reconstructions from *R*~2.5 data without any reordering in time or space dimensions. Figure 8(c) (column) shows the corresponding STCR reconstructions with reordering in both time and space dimensions.

The reordering helps in reconstruction when there is a significant respiratory motion in the data. We previously reported higher acceleration factors (*R*~4 with interleaved undersampling and *R*~5 with variable density sampling) in [3] for myocardial perfusion, when there was minimal or no respiratory motion in the data. In the presence of significant respiratory motion, the method in [3] was not fully able to resolve the artifacts from undersampling. The current method was better able to reduce the artifacts even in the presence of large respiratory motion.

### 4.3. Multi-image brain DTI data

**Figure 9:**
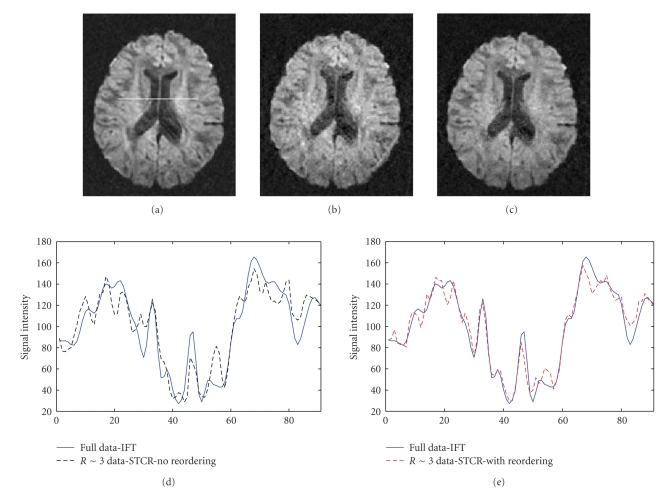
Result of the reordering method on multi-image brain DTI data. (a) Image of a single diffusion encoding direction reconstructed from full Fourier data. A line for comparison of pixel intensity profiles for different reconstructions is also shown. (b) Corresponding encoding direction reconstructed from *R*~3 Fourier data, undersampled in variable density random fashion, using constrained reconstruction in (4) but without any reordering. (c) Corresponding direction reconstructed from the incomplete Fourier data using constrained reconstruction in (4) with reordering. (d) Comparison of intensity line profiles for images in Figures 9(a) and 9(b). (e) Comparison of intensity line profiles for images in Figures 9(a) and 9(c).

The results obtained by applying the reordering method on brain DTI data are shown in Figure 9.

Figure 9(c) matches Figure 9(a) better especially around the ventricular regions. Figure 9(d) compares the line intensity profiles for full data reconstruction (Figure 9(a)) and the reconstruction without reordering (Figure 9(b)). Figure 9(e) compares the intensity profiles for full data reconstruction (Figure 9(a)) and the reconstruction with reordering (Figure 9(c)). The signals in Figure 9(e) match better than those in Figure 9(d).

## 5. DISCUSSION

This paper introduces a modified constraint term for compressed sampling and constrained image reconstruction approaches. In general, it is possible to choose a regularization or constraint term which is a good model for the image being reconstructed. The basic idea of the reordering method is that it is possible to tailor these regularization or constraint operators to improve the reconstruction by reordering the signal. From a compressed sampling point of view, various transforms have been proposed to enforce sparsity in the data. Reordering can be thought as a new set of data-specific “transforms” that further improve the sparsity. Recently, a new method using a prior image constraint [17] was proposed to improve the constrained reconstruction of dynamic CT images. The additional prior image constraint minimizes the *L*_1_ distance between the estimated solution and the prior image. The reordering method proposed here is different in the sense that it does not directly use the intensities in the prior image. The method uses only the ordering information from a prior image or set of images, which can be preserved if the prior images are at a different and unknown intensity scale as compared to the estimated solution.

Reordering can be done in multiple dimensions to improve the sparsity when the signals are not smoothly varying. Here, we used images from the central low-resolution data to determine orderings initially in the multi-image dimension and then used the resulting images to obtain the spatial reordering for each image separately. This is because the central low-resolution data are more faithful in the multi-image dimension than they are in the spatial dimension. Reordering in the multi-image dimension offered more significant improvements as compared to reordering in the spatial dimensions. This is because the temporal constraint generally plays a more important role in resolving the artifacts as compared to the spatial constraint for dynamic imaging [12]. To obtain significant improvements just using spatial reordering, a good high-resolution reference image may be required. Improved ways of obtaining reordering, like doing a separate training scan before the actual acquisition, may help to achieve higher accelerations.

The reordering method incorporates the ordering information of the signal to better match the total variation constraint assumption and thus improves the reconstruction from undersampled data. Methods like adaptive regularization [18, 19] were proposed to improve the performance of TV regularization-based denoising techniques by using a priori information about the signal. In these methods, the regularization parameter is varied based on the a priori knowledge of the locations of edges and smooth regions in a signal, so that less regularization is done where strong edges are present in the signal. While this type of approach may be extended to TV constrained reconstruction, choosing the optimal amount of variation of the regularization parameter can be complicated for rapidly varying signals. An alternative method that can use such a priori information was explored here with the reordering method that uses only a single regularization parameter.

**Figure 10:**
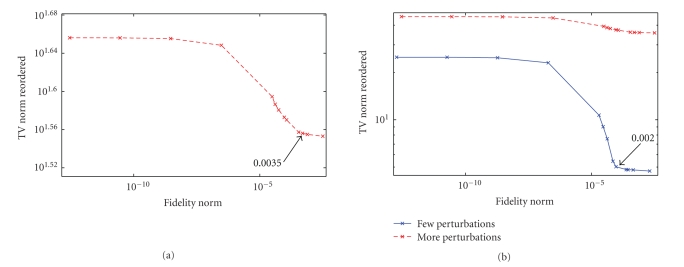
(a) *L*-curve obtained for reconstruction of the nonsmooth signal in Figure 1(b) from *R*~2 Fourier data with reordering, but with large number (~65%) of random perturbations in the exact ordering. (b) *L*-curve obtained for reconstruction of the nonsmooth signal in Figure 1(b) from *R*~2 Fourier data with reordering with fewer (~21%) random perturbations in the exact ordering. The *L*-curve in Figure 10(a) is also overlaid.

The reordering method may not be appropriate when the ordering is incorrect in such a way that the total variation of the reordered full image sets is increased as compared to that of the original full data. In practice, it might not be possible to know this information beforehand. In such cases, *L*-curves can be used to determine to some extent if reordering is appropriate. *L*-curves can be computed for reconstructions with and without reordering and the TV norms corresponding to the optimal regularization parameters can be compared. If the ordering is appropriate, then the TV norm corresponding to the optimal regularization parameter with reordering is lower than that for the corresponding optimal regularization parameter without reordering. Consider Figure 10(a) which shows the *L*-curve obtained for the 1D randomly varying curve shown in Figure 1(b), with a large number of random perturbations (~65%) in the exact ordering. The TV norm corresponding to the optimal *α* is 36.08. When the number of random perturbations is decreased (~21%), the *L*-curve in Figure 10(b) is obtained and the TV norm corresponding to the optimal *α* is 5.02. The *L*-curve in Figure 10(a) is overlaid in Figure 10(b) for reference. The TV norm corresponding to the *L*-curve obtained without reordering is 14.05.

The reconstruction time with image reordering was higher than the standard *L*_1_ norm reconstruction, as in each iteration, the estimated signal is reordered before computing the constraint update. For the data reordering in 1D case, the reconstruction time was 1.04 times slower, while that for the dynamic case with reordering in both spatial and temporal dimensions was 2.8 times slower. It took ~35 seconds per iteration on a linux machine with an AMD processor (2.5 Ghz) and 6 GB ram for STCR with reordering in multiple dimensions. The implementation was done in MATLAB and a host of methods including the use of GPUs are available to greatly speed up reconstruction methods.

## 6. CONCLUSION

A method involving reordering in time and space dimensions of the image estimates to better match the chosen constraints of an inverse problem-type reconstruction was presented. The method uses non-reordered reconstructions to obtain information about the signal to be reconstructed to determine the orderings of the pixel intensities. The orderings can be estimated from the low-resolution images when a variable density undersampling scheme is used, and from non-reordered constrained reconstructions. The method can be forgiving to errors in the images used to choose the orderings as the method does not use the data directly but uses only its ordering information. The method was shown to have promise for cardiac perfusion imaging and offered some small improvements for DTI data. Future improvements in finding more optimal reorderings, perhaps as part of the estimation process, may make the approach useful in a wide array of applications.
